# Effects of saponins Rb_1_ and Re in *American ginseng* combined intervention on immune system of aging model

**DOI:** 10.3389/fmolb.2024.1392868

**Published:** 2024-03-28

**Authors:** Mao Shi, Jie Ma, Shan Jin, Tienan Wang, Yuhan Sui, Lina Chen

**Affiliations:** ^1^ Jilin Provincial Center for Disease Control and Prevention, Changchun, China; ^2^ College of Food Science and Engineering, Changchun University, Changchun, China

**Keywords:** saponin, Rb_1_, Re, aging, immune

## Abstract

Aging is a major risk factor for the development of many pathological processes, such as reduced immunity, cancer, cardiovascular diseases or neurodegenerative diseases, while age-related chronic diseases are the most common causes of death. This paper studies the effects of *American ginseng* saponin Rb_1_ and Re alone and combined intervention on the immune system of aging mouse models, by using 30 mg/kg Rb_1_, 15 mg/kg Re, and Rb_1_ + Re (30 mg/kg Rb_1_ and 15 mg/kg Re (co-intervention) was used to intervene in the aging model, and immune indicators such as thymus index, spleen index, interleukin and interferon were detected to evaluate the impact of Rb_1_ and Re on immune function. The results show that Rb_1_ and Re intervention alone can increase the spleen index by 7%–12% and the thymus index by 12%–19% in the aging model. After Rb_1_ or Re alone intervened, the apoptotic cells in the thymus were slightly reduced, and the proportion of apoptotic cells was reduced. The combination of Rb_1_ + Re can promote the thymus index and spleen index to increase by 23.40% and 25.5% respectively, which is more advantageous than Rb_1_ or Re alone. In addition, Rb_1_ and Re intervention can reduce the level of interferon INF to a level comparable to that of young mice. Rb_1_ + Re can not only reduce the INF content, but also reduce the TNF content. The above results show that *American ginseng* saponin Rb_1_ and Re can delay the decline of the immune system in the aging model, and the combined intervention of the two is significantly better than individual intervention in the recovery of the immune system. This paper can provide theoretical basis and data support for the development of *American ginseng* nutritional supplements and its application in aging groups products to improve immunity.

## 1 Introduction

*American ginseng* (*Panax quinquefolius L., Araliaceae*) is a perennial herb of the genus Panax and is native to deciduous woodlands in eastern North America (Szczuka et al., 2019). *American ginseng* is grown in many parts of the world, and the main producing areas are in North America, such as Wisconsin, Michigan, North Carolina, Ohio, Tennessee, and Ontario and British Columbia in Canada (Liu et al., 2022); It is also grown commercially in China and South Korea (Qiang et al., 2020). The stems, leaves and roots of *American ginseng* are both effective, but the roots are more commonly used as medicine (Punja, 2011). *American ginseng* has been shown to be effective in treating anxiety (Bell et al., 2022), regulating blood pressure (Vuksan et al., 2019), enhancing immunity (Liu et al., 2020), lowering blood sugar (Zhang et al., 2023), reducing inflammation (Liu et al., 2019), protecting cardiovascular system (Zhou et al., 2023), etc., and has a wide range of application precedents. As a traditional medicine, dietary supplement, functional food and beverage, and natural cosmetic additive, *American ginseng* is also widely used in healthcare products, food and cosmetics (Leung and Wong, 2010). In addition, studies have shown that the extract of *American ginseng* can play an obvious anti-feeding effect on insects (Shou-rong et al., 2022), so the extract can be applied to agriculture and other fields. *American ginseng* has been more and more recognized in the world, because it has a higher cost performance, and has been more widely promoted in the fields of biomedicine, food healthcare, chemical industry and so on.

The most important component of *American ginseng* is saponin, which is also the most important component of *American ginseng* to play the drug effect. Saponins and non-starch polysaccharides in *American ginseng* have been proved to play a positive role in immune regulation, oxidation regulation, blood glucose regulation, anti-radiation and other aspects (Bell et al., 2022). In addition, *American ginseng* also contains a small amount of phenols (Szczuka et al., 2019), flavonoids, volatile oils, vitamins and minerals (Cui et al., 2017). The saponins in *American ginseng* are generally named “Rx,” where “R” represents the root, “x” describes the polarity of the compound in alphabetical order, and are named “Ra,” Rb……“Rh” , according to their mobility on the TLC plate (Feng et al., 2017).

Most saponins are arranged by 17 carbon atoms and form four damarane rings. According to the different number of hydroxyl groups, saponins can be divided into two categories: propanaxanediol (PPD) and propanaxantriol (PPT) (L et al., 2021). Generally, Rb_1_, Rb_2_, Rb_3_, Rc, Rd, Rg_3_, and Rh_2_ belong to PPD. Re, Rf, Rg_1_, Rg_2_, Rh_1_, F1, and F3 belong to PPT. Among the main components, Rb_1_, Re, Rd, Rg_1_, and Rb_3_ accounted for more than 70% of the total saponin content. Different saponin components have different functions, some of which have been shown to inhibit cancer cell growth, antioxidant, neuronal repair, stabilize the heart, lower blood sugar levels, activate estrogen receptors, improve memory, and so on. In addition, the main components of saponins in *American ginseng* are very close to ginsenosides, so *American ginseng* is usually compared with ginseng. In the theory of traditional Chinese medicine, *American ginseng* has mild drug properties while ginseng has mild drug properties (Li et al., 2021). Therefore, western ginseng has similar effects in many ways. Daria Szczuka (Szczuka et al., 2019) summarized the major ginsenosides of *American ginseng* and their pharmacological activities (Table 1).

**TABLE 1:** Main ginsenosides of *American ginseng* and their pharmacological activities.

Saponin component	Pharmacological action
Rb_1_, Rb_2_, Rc	The synthesis of acetylcholine in hippocampus was induced by stimulating choline acetyltransferase Zhang et al. (2023b). Reversible and tetanically block voltage-dependent Na + channels in the brain, reducing the harmful effects of hypoxia Mancuso and Santangelo (2017). Downregulated COX-2 gene; Stable neutrophils and lymphocytes; Inhibit the release of histamine; Block calcium channels and stabilize the heart; Lower blood sugar levels; Anti-diabetes, insulin sensitization and anti-obesity effects Liang et al. (2019). Neuroprotective, neuroprotective, estrogen-like activity Yao and Guan (2022). Stimulates GABA receptors and induces inhibitory effects on brain function, which highlight its sedative, anti-anxiety, sleep, relaxation, and antipsychotic effects Jiao et al. (2016)
Rb_2_	Stimulating superoxide dismutase, Inhibit angiogenesis in cancer, Prevention of diabetes Zhang et al. (2023b). Lowers cholesterol and triglyceride levels, activates lipolysis Liang et al. (2019). Corticotropic hormone and estrogen activity Yao and Guan (2022)
RC	Inhibit the proliferation of breast cancer cells Liang et al. (2019); Induce corticotropic effects Jiao et al. (2016)
Rd, Rc, Re	Promote neurite growth, which is an important process of neuronal repair Rokot et al. (2016). Induce corticotropic effects Razgonova et al. (2019)
Re	Scavenging hydroxyl radical and degrading H2O2 Zhang et al. (2023b). Lower blood sugar levels Liang et al. (2019). Induce cardioprotective effect Yao and Guan (2022). Activates cGMP and relaxes smooth muscle Razgonova et al. (2019)
Rg_1_	Downregulated COX-2 gene; Stable neutrophils and lymphocytes; Inhibition of histamine release Zhang et al. (2023b); Inhibition of thromboxane activation induced by platelets, Increased insulin receptors, Increased T helper lymphocytes Jiao et al. (2016). Inhibition of endothelin release and relaxation of vascular smooth muscle, Activate cyclic guanosine phosphate and relax smooth muscle (antihypertensive effect) Rokot et al. (2016). Block calcium channels and stabilize the heart, Lower blood sugar levels Razgonova et al. (2019)
Rg_2_	Inhibitory neuron acetylcholine Jiao et al. (2016)
Rg_3_	Inhibit thrombine-induced platelet aggregation, Relax the smooth muscle of blood vessels by activating K+ channels and releasing Ca 2+ Zhang et al. (2023b). Inhibit tumor progression and reduce drug resistance of cancer cells, Inhibition of endothelin and vascular smooth muscle relaxation Mancuso and Santangelo (2017). Cause the effect of lowering blood pressure, Downregulated COX-2 gene; Stable neutrophils and lymphocytes Jiao et al. (2016). Inhibition of histamine release Razgonova et al. (2019). Regulates mitogen-activated protein kinase, thereby inhibiting the spread of cancer cells Popovich and Kitts (2004)
Rh_1_	Activation of estrogen receptors Jiao et al. (2016). Inhibit the proliferation of cancer cells and induce apoptosis Popovich and Kitts (2004)
Rh_2_	Inhibit the proliferation of breast cancer, liver cancer and prostate cancer cells Popovich and Kitts (2004)

The total saponins of *American ginseng* are a slow accumulation process in the roots of *American ginseng*. It has been reported that the content of Rb_1_ in total saponins of *American ginseng* is the highest, followed by Re, Rd, Rc, and Rg_1_ (Yang et al., 2015). In addition, it has been reported that F11 contains the least amount of saponins in *American ginseng* (Piao et al., 2020). Although the types of saponins in *American ginseng* and ginseng are very similar, there are still big differences between them. The Rg_1_ content of *American ginseng* is much lower than that of ginseng, while the Rb_1_ content is close to that of ginseng (Zhao et al., 2006).

The immune system is the body’s natural defense against external stimuli. The immune system’s response to stimulus signals is extremely complex and precisely coordinated to protect the body from pathogens and maintain homeostasis. The immune response depends on B lymphocytes (humoral immunity) and T lymphocytes (cellular immunity), respectively. Studies have shown that *American ginseng* and jujube extracts can significantly enhance the immune function of ICR mice, mainly manifested in increased serum hemolysin production and accelerated T cell proliferation in the administration group (Yu et al., 2016). Rajarshi Ghosh et al. extracted non-starch polysaccharide AGC3 from the cell culture medium of *American ginseng*, and observed that AGC3 could significantly enhance the activities of IL-6, TNF-α, GM-CSF and MCP-1 in mouse macrophages and primary mouse spleen cells (Ghosh et al., 2020). Natural killer (NK) cells play an important role in the innate immune system, and NK cells can recognize tumor cells and virus-infected cells as non-self elements by recognizing MHC Class I autoantigens. In the secondary lymphoid tissue of the spleen, tonsils and lymph nodes, about 5%–20% of the lymphocytes are NK cells, accounting for a high proportion. Studies have shown that adding ginseng extract CVT-E002 to mouse diets can effectively increase the number of NK cells in mice (Miller et al., 2012). Clinical experiments have also shown that water-soluble extract of ginseng can enhance NK cell activity (Kang and Min, 2012) Zubair’s research has shown that ginseng also has antiviral and antifungal effects, and can be used as an adjuvant in some drugs and vaccines to enhance the effect (Ratan et al., 2021). In summary, ginseng extract has many effects on immunity, and in addition to saponins, some polysaccharides and water-soluble extracts can also greatly promote positive immune response.

With the growth of individual age, usually the body’s immune function will be severely damaged or even deteriorated, this process is also known as immune aging. It is characterized by a general disruption of immune homeostasis, including impaired development of immune cells in the bone marrow, degeneration of the thymus, increased autoimmune risk, and a weakened response to new and chronic infections (Babayan et al., 2018). In addition, the dysfunction of the immune system is an important sign of aging. Among all immune organs, the thymus is a central lymphoid organ responsible for the production of original T cells, which plays a crucial role in mediating cellular and humoral immunity (Thapa and Farber, 2019). Chronic degeneration of the thymus with age is considered to be one of the main factors leading to the loss of immune function (Wei et al., 2015). The spleen is usually involved in the regulation of humoral immunity (Abdallah and Hassanin, 2015). Both the thymus and spleen are thought to be closely involved in the aging of the body. Therefore, the evaluation of thymus and spleen is an important index to evaluate the immune function of the body. In addition, the most significant changes in immune aging include severe loss of initial T cells and accumulation of memory T cells, reduced CD4/CD8T cell ratio and number of B cells, and upregulation of circulating pro-inflammatory cytokines, especially IL-6 and TNF-α (Rais et al., 2017). The occurrence of inflammation and aging is a complex formative process, and inflammation levels generally increase with age even in the absence of acute infection or other physiological stress (Nelsen et al., 2014). In the context of inflammation and aging, ROS is often the main molecule that accelerates inflammation related tissue degradation and aging. Evidence suggests that aging, oxidative stress (Cavazzana et al., 2017), and inflammation are interdependent (Milner, 2018).

Aging is a major risk factor for the development of many pathological processes, especially the most significant problem associated with aging is the body’s reduced immunity, which leads to cancer, cardiovascular disease or neurodegenerative diseases, and age-related chronic diseases are also the most common cause of death. Delaying aging has become the goal of sociology and gerontology. Aging is usually accompanied by decreased immunity, intestinal and immune changes, so how to delay aging and improve the quality of life of aging population is a worthy research topic. However, the researches on senescence of *American ginseng* are few and incomplete. In order to more comprehensively study the effects of *American ginseng* saponins on the immune system of aging models, this study prepared monomeric saponins, fed aging mice, and evaluated the effects of Rb_1_ and Re alone or combined intervention on the levels of thymus index, spleen index, thymus apoptotic cells, IgA, IgG, and IgM in aging models. In order to provide theoretical basis and data support for the development of *American ginseng* nutritional supplements.

## 2 Materials and methods

### 2.1 Materials and reagents

Pharmaceutical grade saponins Rb_1_ and Re were purchased from the Natural Medicine Chemistry Laboratory of Jilin University and stored at 4°C according to the drug instructions. Aging model mice (C57BL/6.18 months old) were purchased from Changchun Yishi Experimental Animal Technology Co., LTD., and raised in Jilin Provincial Health Inspection Center {approved by the Experimental Animal Management Committee of Jilin Provincial Center for Disease Control and Prevention [JCDC (2023) No. 1]}. Mouse T lymphocyte population (CD3, CD4, CD8) ELISA kit, Shanghai Varan Biological Company. Mouse interleukin INF ELISA kit, Mouse interleukin TNF ELISA kit, Mouse interleukin 27 (IL-27) ELISA kit, Mouse interleukin 17 (IL-17) ELISA kit, Mouse interleukin 10 (IL-10) ELISA kit, Mouse interleukin 6 (IL-6) ELISA kit, Mouse interleukin-4 (IL-4) ELISA kit, Mouse interleukin 2 (IL-2) ELISA kit, All purchased from Shanghai Enzyme Research Biology. Mouse immunoglobulin G (IgG) test kit, Mouse immunoglobulin M (IgM) test kit, Mouse immunoglobulin A (IgA) test kit, All purchased from BioLegend.

### 2.2 Grouping and administration of experimental mice

Forty healthy male C57BL/6 mice (18 months old, weight 28–32 g) were selected. The aging mouse models were put into the mouse house for 7 days, then they were divided into 4 groups on average, with 10 mice in each group. They were divided into Aged model group, Rb_1_ administration group (Rb_1_), Re administration group (Re) and Rb_1_, Re administration group (Rb_1_ + Re). The dosage was formulated according to the optimal concentration of the team’s previous trial, that is, the dosage of Rb_1_ was 30 mg/kg, and the dosage of Re was 15 mg/kg. In addition, 10 6-month-old male healthy C57BL/6 mice (weight 20–25 g) were used as a control group (Col).

The mice were raised in Jilin Provincial Health Inspection Center in a constant temperature (21°C ± 2°C) and 50% humidity environment, and the light was set to 12 h dark light cycle, and they were free to drink and eat. The saponin monomer drug was dissolved in distilled water according to the experimental design, and the mice were given the drug through drinking water every day. Control and aging groups drank distilled water. Before feeding, each mouse was starved for 12 h and then numbered and weighed. After 8 weeks of continuous feeding, each mouse was starved for 12 h, and then weighed. The mice were killed 20 h after the last administration and the relevant indexes were detected.

### 2.3 Laboratory mouse anatomy

The mouse model was anesthetized with 0.5% pentobarbital sodium, measured the body length, removed the eyeball and collected blood. The blood was left for 15 min, centrifuged at 3,000 r/min at 4°C for 10 min, and the upper serum was collected and placed in the EP tube at −80°C for freezing. At the same time, the heart, liver, spleen, lung, kidney and thymus tissues were quickly washed in pre-cooled normal saline, dried by filter paper and weighed, part of them were fixed in 4% paraformaldehyde solution to prepare paraffin sections, and part of them were placed in frozen tubes and put into liquid nitrogen, and frozen at −80°C to be measured. The remaining biological tissues after dissection were treated harmlessly.

### 2.4 Thymus index, spleen index and body weight growth rate statistics

Electronic balance is used to weigh the thymus and spleen to the exact milligram. The thymus index and spleen index are calculated according to the Eqs 1, 2.

$$\text{Thymus index }\%=\frac{\text{Thymus}\;\text{weight}\;\left(\text{mg}\right)}{\text{Weight}\;\left(g\right)\times 1000}\times 100\%$$ (1)

$$\text{Spleen index }\%=\frac{\text{Spleen}\;\text{weight}\;\left(\text{mg}\right)}{\text{Weight}\;\left(g\right)\times 1000}\times 100\%$$ (2)

The weight growth rate of each mouse was calculated by the Eq. 3:

$$\text{Weight growth rate }\%=\frac{W_{2}\left(g\right)-W_{1}\left(g\right)}{W_{1}\left(g\right)}\times 100\%$$ (3)

Where W_1_ was the weight before treatment and W_2_ was the weight after 8 weeks of administration.

### 2.5 Thymus cell culture and apoptosis detection

After the mice were anesthetized and killed, the thymus tissue was dissected, and cell suspension was prepared using a tissue fragmentation apparatus, and the cell concentration was adjusted to 5 × 10^6^/mL. The cells were washed with PBS for 3 times and cultured with RPMI 1640 medium. After overnight culture, the culture solution was removed, cleaned with PBS for 3 times, and then centrifuged at 1,500 r/min for 5 min. Then RNase was added and incubated in a metal bath at 37°C for 30 min. Then PBS was used to clean it three times, TUNEL dye solution and tritonX-1000 were added and stored at 4°C for 30 min away from light. Then, all cells were stained with H33342, and after adding H33342 dye solution, they were stored at 4°C for 30 min away from light. They were then observed under a fluorescence microscope. In the visual field, green is apoptotic cells and blue is all cells. The overall state of thymus was judged according to the proportion of apoptotic cells.

### 2.6 Blood IgG assay

After blood coagulation, mouse serum was obtained by centrifugation at 3,000 r/min for 10 min and stored at −20°C until use. According to the kit instructions, the mouse immunoglobulin G (IgG) test kit was used to determine the total IgG concentration in mouse serum. The brief steps are as follows: (Szczuka et al., 2019): Take a sufficient number of enzyme-labeled coated plates, set standard product holes, sample holes to be measured and blank control holes, and add standard product 50 μL into the standard product holes; Add 10 μL of the sample to be measured in the sample hole, and then add 40 μL of the sample diluent (that is, the sample dilution is 5 times); Blank control hole is not added; (Liu et al., 2022); Incubate in a 37°C incubator for 30 min, Discard the liquid, pat dry on the absorbent paper, fill each hole with washing liquid, leave for 1 min, shake off the washing liquid, pat dry on the absorbent paper, repeat washing board 4 times; (Qiang et al., 2020); Add 50 μL of enzyme-labeled working liquid, and do not add the blank control hole; (Punja, 2011); Incubate in a 37°C incubator for 30 min, discard the liquid, pat dry on absorbent paper, fill each hole with washing liquid, leave for 1 min, shake off the washing liquid, pat dry on absorbent paper, repeat washing board 4 times; (Bell et al., 2022); Each well was first added with developing agent A 50 μL, then with developing agent B 50 μL, and developed for 15 min at 37°C away from light; (Vuksan et al., 2019); Remove the enzyme label plate, add 50 μL termination solution to each hole, terminate the reaction, adjust the zero with blank holes, and measure the light absorption value of each hole with 450 nm wavelength within 15 min after termination; (Liu et al., 2020); According to the concentration of the standard product and the corresponding OD value, the linear regression equation of the standard curve is calculated, and then the corresponding sample concentration is calculated on the regression equation according to the OD value of the sample. The final concentration is the actual measured concentration multiplied by dilution.

### 2.7 Blood interleukin and interferon assay

Serum was isolated according to the method described in 2.6. The content of interleukin was determined according to the kit instructions. The brief steps are as follows: (Szczuka et al., 2019): The number of slats required was calculated in advance, and 30 min before the experiment, the kit was taken out, restored to room temperature, 100 μL standard working liquid and test sample were added to each reaction hole, the standard product was repeated three times, the plate was sealed and incubated at 37°C for 90 min, the liquid was discarded and dried. 100 μL biotin-labeled interleukin antibody was added to each reaction hole, and the plates were sealed and incubated at 37°C for 60 min (Liu et al., 2022). Discard the liquid, shake dry, add 350 μL washing solution to each reaction hole, soak for 1–2 min, shake dry the washing solution, repeat 3 times; (Qiang et al., 2020); Each reaction hole was added with 100 μL HRP labeled streptavidin and incubated at 37°C for 30 min after plate sealing; (Punja, 2011); Add 300 μL washing liquid to each reaction hole, and shake the washing liquid dry at an interval of 30 s. Repeat 4 times; (Bell et al., 2022); Add 90 μL color developer (dark) to each reaction hole, and hide color from 37°C for about 15 min after sealing the plate; (Zhang YT. et al., 2023); Add 50 μL termination solution into each reaction hole, and immediately measure OD value with enzyme-labeled instrument at 450 nm wavelength (Within 5 min), calculate the average OD value of the standard and sample: the OD value of each standard and sample should be subtracted from the OD value of the blank hole. The content of interleukin in serum samples was calculated according to the standard curve.

### 2.8 Lymphocyte subpopulation analysis

The collected fresh blood was added with heparin sodium for anticoagulation, and then centrifuged at 1,700 rpm for 10 min to separate the serum. After discarding the supernatant, 200 μL normal saline was added to reinsert the cells. 1 mL of Ficoll lymphocyte separation fluid was added to the new centrifuge tube, and then the resuspended whole blood cells were slowly added so that the cells were on top of the lymphocyte separation fluid, while the fluid level was kept stratified. After centrifugation at low temperature (1,200 rpm, 30 min), the centrifugation end page was divided into 4 layers, in which peripheral blood mononuclear cells (PBMC) were in the second layer, and the PBMC layer was completely absorbed by pipetting gun to obtain relatively pure PBMC. 2 mL normal saline was added to the extracted PBMC, then centrifuged at 1,700 rpm for 10 min, and the supernatant was discarded to obtain PBMC precipitation. PBMC precipitation was re-suspended in RPMI l640 medium with 50 μL. Appropriate amount of cell suspension was added to the cell incubation board, leukocyte combination stimulant was added to each hole, and the mixture was blown and cultured in the incubator for 1 h, and then protein transport inhibitor containing Monensin was added to the incubation hole, and the culture was continued for 3 h. After taking out the cell incubation plate, blow and mix all the liquid in the hole, transfer it to the flow tube as much as possible, add 1 mL PBS to mix, centrifuge at 1,200 rpm for 5 min, and discard the supernatant. 2 μL cell surface cytokine fluorescently labeled antibody was added to the detection tube, and isotype control antibody was added to the care, and incubated at 4°C for 30 min in the dark. Then 1 mL PBS solution was added into the flow tube, blown and mixed well, centrifuged at 1,200 rpm for 5 min, and the supernatant was discarded. Each tube was fixed with 4% paraformaldehyde, and then cleaned with 1 mL PBS solution. During the cleaning process, blow and mix well, then centrifuge at 1,200 rpm for 5 min, and discard the supernatant. 200 μL of film breaking liquid was added into each flow tube and placed at 4°C away from light for 20 min. Next, add 1 mL washing TM buffer to wash, blow and mix well during washing, then centrifuge at 1,200 rpm for 5 min, discard the supernatant, and repeat cleaning twice. Finally, the cells were suspended with 100 μL TM buffer and added cytokine antibodies, incubated at 4°C for 30 min in the dark, then centrifuged at 1,200 rpm for 5 min, the supernatant was discarded, and 400 μL PB solution was used for re-suspension precipitation, and flow detection was performed in flow cytometry.

### 2.9 Data processing and analysis

SPSS 19.0 was used for statistical analysis. The two sets of data were directly compared with the practical one-way ANOVA or T-test. Three or more sets of data were compared for analysis using ANOVA. Data are expressed as mean ± standard deviation of no less than 3 biological replicates. The significance level was significant with *p* < 0.05.

## 3 Results

### 3.1 Effects of different saponin monomers on thymus index, spleen index and body weight growth rate

After 8 weeks of drug administration, the mice were dissected and their internal organs were observed. The results showed that saponin monomer administration had a positive effect on the aging model. Administration of Rb_1_ and Re alone produced different effects than simultaneous administration of Rb_1_ + Re. Saponins had no significant effects on the heart, liver, lung and kidney. But it has positive effects on the spleen, thymus and liver. After analysis, the spleen index and thymus index were significantly increased in the saponin administration group.

**FIGURE 1:**
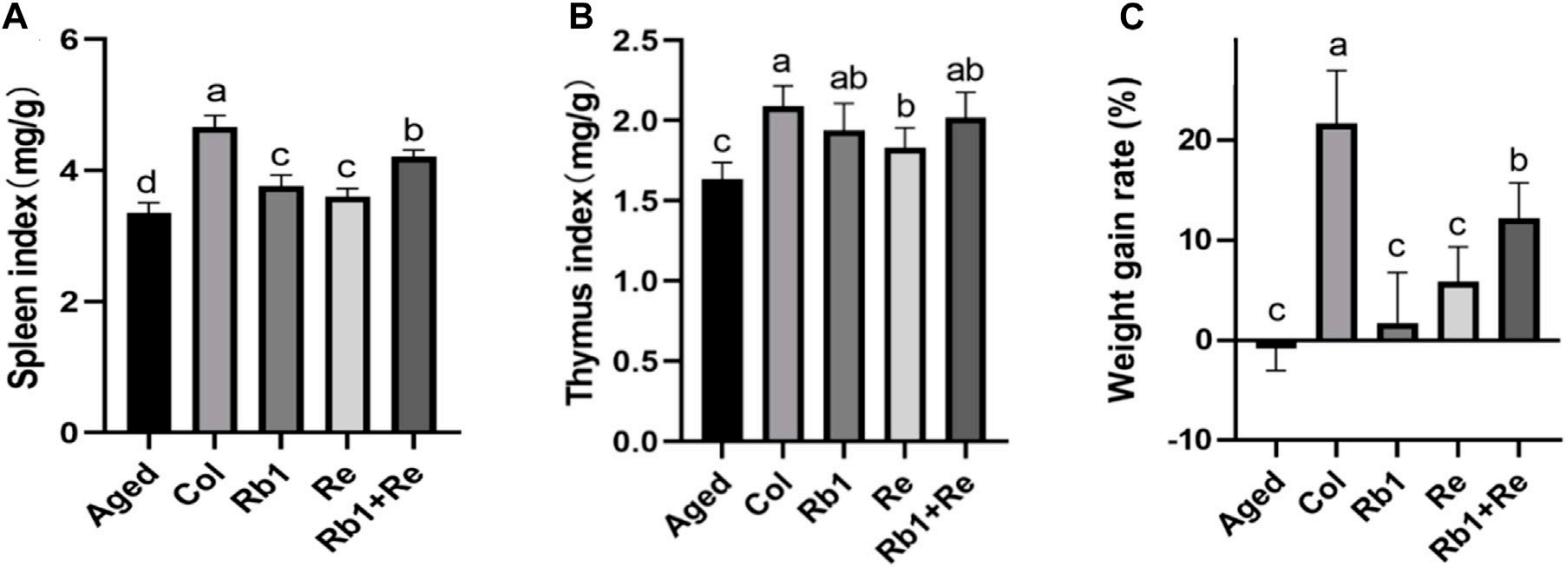
Effects of different saponin monomers on thymus index, spleen index and body weight growth rate **(A)** Effects of difference dose of Re on the spleen index of aged mice. **(B)** Effects of difference dose of Re on the thymus index of aged mice. **(C)** Effects of difference dose of Re on the weight gain of aged mice. Note: Identical letters indicate the absence of a significant difference (*p* > 0.05) and different letters indicate a significant difference (*p* < 0.05).

The results of spleen index showed that the spleen index of Col group was the highest and significantly higher than that of aging model group. Rb_1_ and Re administration alone can significantly increase the spleen index in aging models, ranging from 7% to 12%. Combined administration of Rb_1_ + Re showed a higher spleen index than administration of Rb_1_ or Re alone, with Rb_1_ + Re leading to a 25.5% increase in spleen index. However, the thymus index in all treatment groups was significantly lower than that in Col group (Figure 1A). Accordingly, the combination of Rb_1_ and Re has a more significant effect on spleen index, which may be caused by the superimposed effect of Rb_1_ and Re.

The thymus index of Col group was the highest and significantly higher than that of Aged group. After Rb_1_ and Re monomer treatment, the thymus index was significantly higher than that of Aged group. After analysis, compared with Aged group, the thymus index in Rb_1_ group was 18.67% higher, that in Re group was 12.12% higher, and that in Rb_1_ + Re combined therapy was 23.40% higher. However, there was no significant difference between all groups. In addition, the thymus index in the Re group was significantly higher than that in the Aged group, but significantly lower than that in the Col group. However, the Rb_1_ + Re group showed no significant difference from the Rb_1_ administration group and Col group (Figure 1B). Therefore, both Rb_1_ and Re can have a positive effect on thymus index, and Rb_1_ has a greater effect than Re.

By analyzing the effects of Rb_1_, Re and Rb_1_ + Re on the weight growth rate of aging models, this study found that Rb_1_ and Re alone can significantly increase the weight growth rate, but the growth rate is far less than that of the control group. However, combined administration of Rb_1_ + Re significantly increased the growth rate of body weight compared with administration of Rb_1_ or Re alone, by 5.96 times and 1.08 times (Figure 1C). In addition, the weight growth rate of all groups was significantly higher than that of Aged group.

### 3.2 Effects of different saponin monomers on apoptosis of thymus cells

**FIGURE 2:**
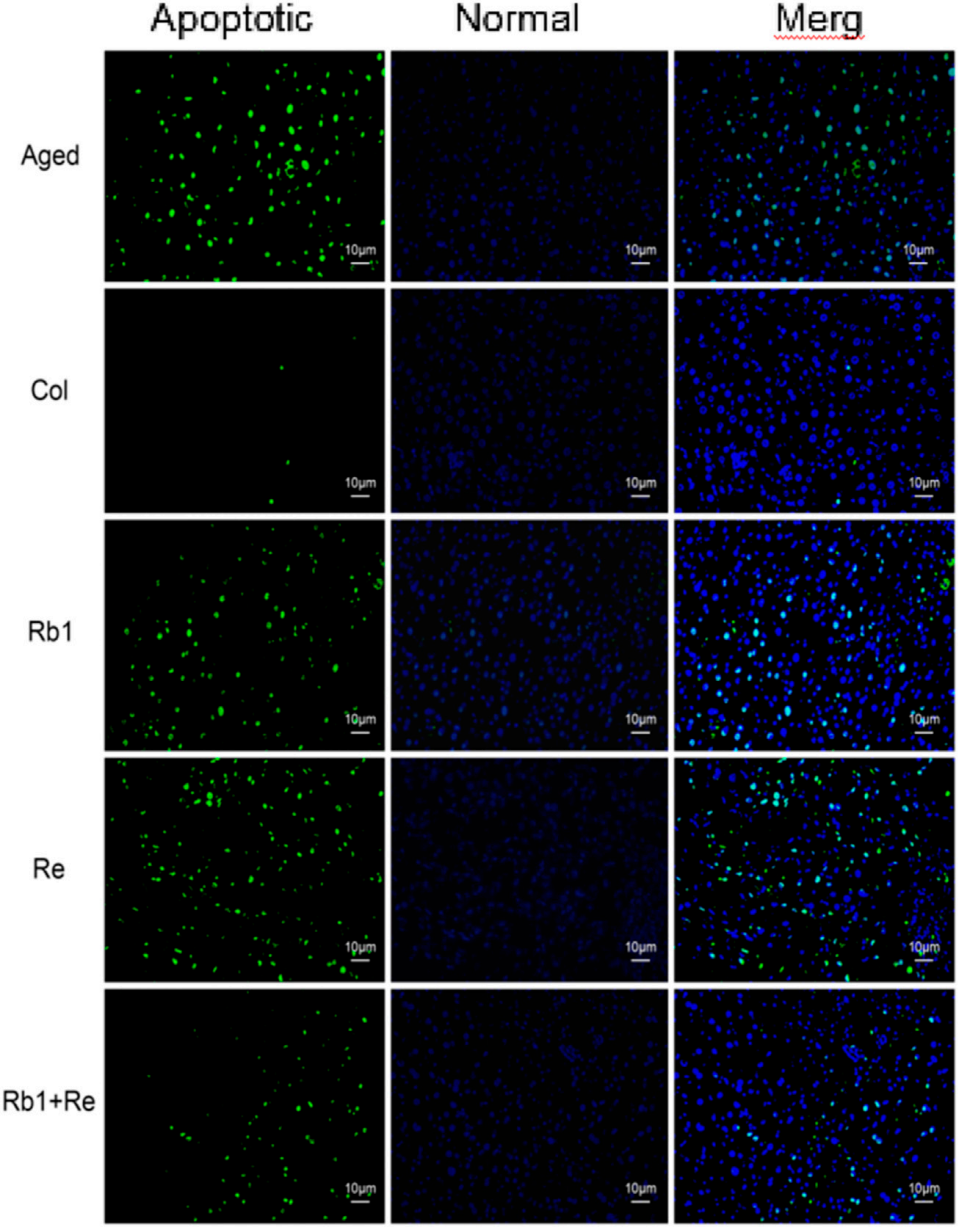
Effect of different saponin monomers on thymocyte apoptosis.

The macroscopic condition of thymus can be evaluated by detecting apoptotic cells of thymus. The results showed that there were a large number of apoptotic cells in the aging mouse model, but fewer apoptotic cells in the Col group. However, after the intervention of Rb_1_ or Re alone, the apoptotic cells of thymus decreased slightly, and the proportion of apoptotic cells decreased. It is worth noting that apoptotic cells in the thymus were significantly reduced after the combined intervention of Rb_1_ and Re, which indicates that the combined intervention of Rb_1_ and Re has a alleviating effect on thymocytes in the senescent state (Figure 2). This is in good agreement with the thymus index. However, after the joint intervention of Rb_1_ and Re, the thymocytes still could not restore to the Col group state. This may be due to the fact that saponin monomers can remove oxidative free radicals in aging body, maintain REDOX balance, and promote the growth of thymus cells (Rais et al., 2017). In addition, the results of this study also suggest that Rb_1_ and Re combined use has a stronger immune-enhancing effect than the single use.

### 3.3 Effects of different saponin monomers on IgA, IgM and IgG in blood

**FIGURE 3:**
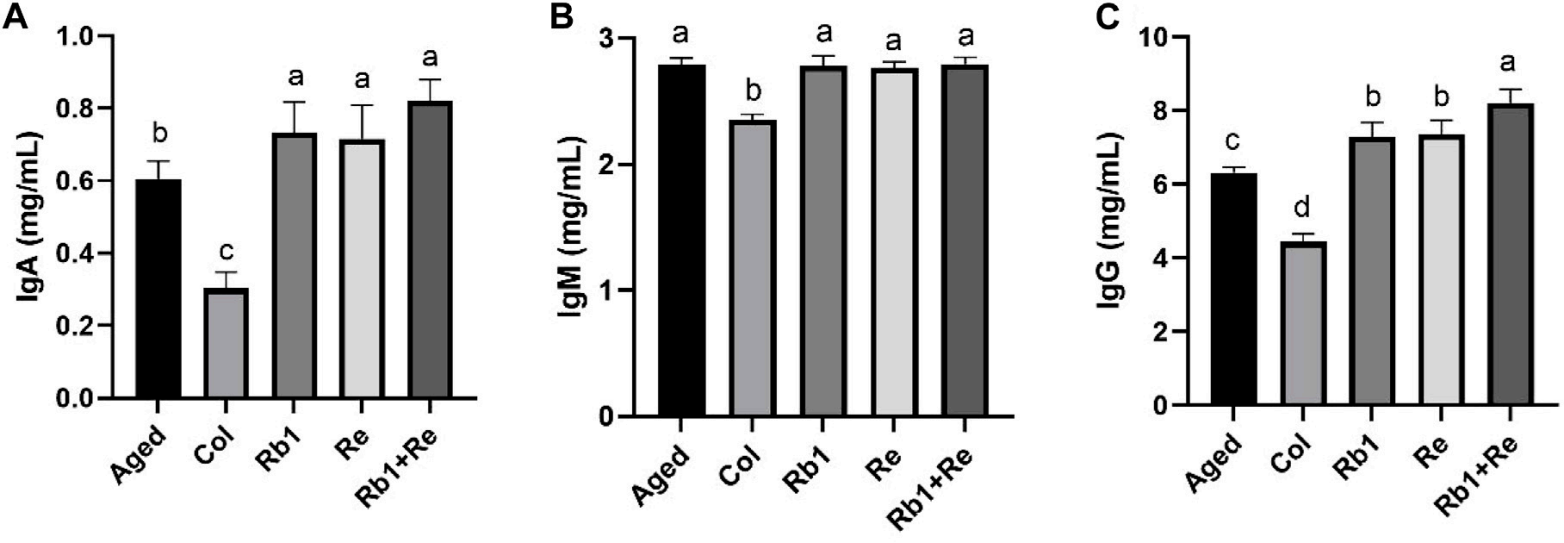
Effects of difference dose of Re on the immunoglobulin level of aged mice. **(A)** Effects of difference dose of Re on IgA level of aged mice. **(B)** Effects of difference dose of Re on IgM level of aged mice. **(C)** Effects of difference dose of Re on IgG level of aged mice. Note: Identical letters indicate the absence of a significant difference (*p* > 0.05) and different letters indicate a significant difference (*p* < 0.05).

It is well known that immune antibodies come from T cells and B cells. They recognize antigens produced by external microorganisms or toxins (Nelsen et al., 2014). Once the antibody finds the intruder, the cell quickly defends itself against the threat. There are five main immunoglobulin (Ig) antibodies in the body: IgA, IgG, IgM, IgD, and IgE (Cavazzana et al., 2017). These antibodies are all produced by B cells. Among them, IgA, IgM, and IgG are commonly used for diagnosis. When our body is infected by an organism, our immune system rapidly produces IgA and IgM and acts as the earliest antibodies to defend against or delay the infection (Milner, 2018). Within a few weeks, our immune system produces IgG, while IgA and IgM gradually break down. Thus, IgA and IgM work in the short term, while IgG is involved in the long term response. The results of this study showed that the levels of IgA, IgM, and IgG in aging model mice were significantly higher than those in control group. However, when saponin monomer was administered, IgA and IgG contents increased significantly, but IgM contents did not show significant changes. There was no significant difference between Rb_1_ administration alone and Re administration alone. However, when Rb_1_ and Re were administered simultaneously, IgG levels in aging mouse models were significantly increased (Figure 3). Different from the aging model mice, the levels of various immunoglobulins in the Col group were lower, which also indicated that the inflammation of the Col group mice was lower.

At the same time, the results of this study also showed that after 8 weeks of administration, *American ginseng* monomer Rb_1_ and Re produced a long-term response to the aging model, so the level of IgA and IgM increased limited, while IgG increased significantly. In particular, IgA content increased significantly after administration, but the increase was small, while IgM did not change significantly, so the effect of *American ginseng* monomer Rb_1_ and Re on the aging model is long-term. In addition, simultaneous administration of Rb_1_ and Re produced significantly better effects than single administration.

### 3.4 Effects of different saponin monomers on blood interleukin and interferon

**FIGURE 4:**
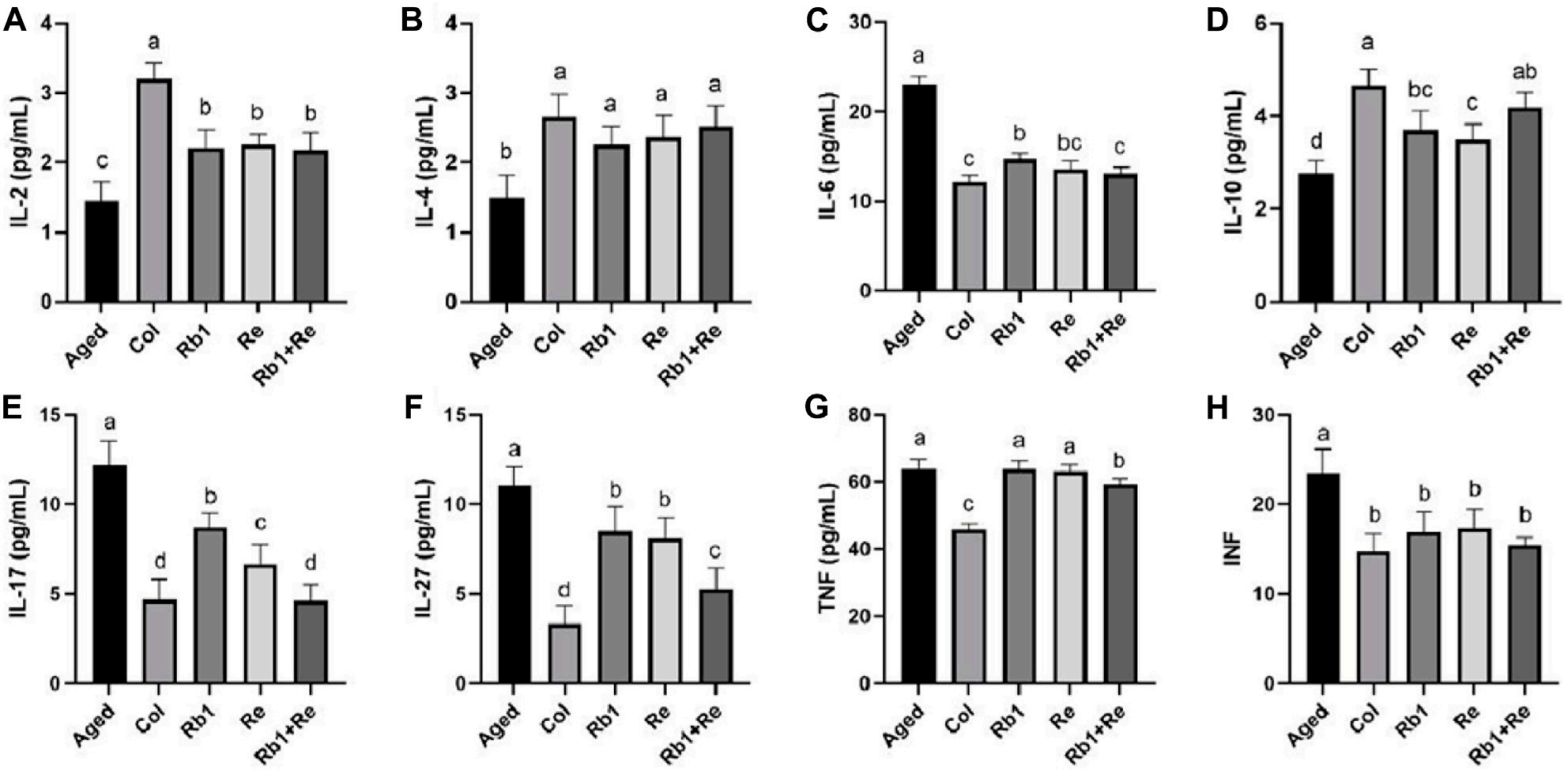
Effects of difference dose of Re on the blood interleukin and interferon of aged mice. **(A)** Effects of difference dose of Re on IL-2 level of aged mice. **(B)** Effects of difference dose of Re on IL-4 level of aged mice. **(C)** Effects of difference dose of Re on IL-6 level of aged mice. **(D)** Effects of difference dose of Re on IL-10 level of aged mice. **(E)** Effects of difference dose of Re on IL-17 level of aged mice. **(F)** Effects of difference dose of Re on IL-27 level of aged mice. **(G)** Effects of difference dose of Re on TNF level of aged mice. **(H)** Effects of difference dose of Re on INF level of aged mice. Note: Identical letters indicate the absence of a significant difference (*p* > 0.05) and different letters indicate a significant difference (*p* < 0.05).

The results of this study showed that the contents of IL-2, IL-4, and IL-10 in the Col group were significantly higher than those in the Aged group, but the corresponding levels of IL-2, IL-4, and IL-10 in the Aged group were significantly increased after Rb_1_ and Re treatment. In particular, IL-4 levels in the aging model were not significantly different from those in the Col group after Rb_1_ and Re administration. When Rb_1_ and Re were administered at the same time, the content of IL-10 increased to a level close to Col. In addition, IL-6, IL-17, and IL-27 concentrations were found to be highest in the aging model, but lowest in Col. However, after administration of Rb_1_, Re or Rb_1_ + Re at the same time, its content decreased significantly, and the effect of simultaneous administration was the most obvious. At the same time, interferon TNF and INF were measured in this study, and the results showed that the aging model had higher levels of TNF and INF than the control group. After administration, INF levels returned to levels comparable to Col, but TNF was not affected. Simultaneous administration of Rb_1_ and Re significantly reduced TNF levels in the aging model mice, but they were still much higher than those in the Col group (Figure 4).

The thymus and spleen are the main places of immune function and the natural barrier of defense against invasion. Thymus index and spleen index can reflect the immune level of the body to a certain extent (Leite-De-Moraes et al., 2001). Studies have shown that aging can inhibit the proliferation of immune cells and impair the development of immune organs, thus affecting the immune response ability of the body (Onodera et al., 2017). However, drug intervention can have positive effects on thymus index and spleen index. The results of this study show that the thymus index and spleen index of the Aged group model are significantly lower than those of the Col group, which indicates that the immune organs of the aged model have certain atrophy, which also indicates that their immunity is declining. After 8 weeks of Rb_1_ and Re intervention, the thymus index and spleen index of the aging model were significantly higher than those of the Aged group, but lower than those of the Col group, indicating that saponin Rb_1_ and Re had a positive effect on the immune organs of aging mice, but the thymus index and spleen index could not be restored to the level of Col group by administration of Rb_1_ and Re. In addition, the combination of Rb_1_ and Re produced a better effect than the administration alone. In addition, by measuring the body weight growth rate, it was found that the combined administration of Rb_1_ + Re could effectively increase the body weight growth rate, which indicated that the body capacity of the aging model was well restored.

## 4 Discussion

Immunoglobulin is one of the non-specific immune factors of the body, and it is the basic index to evaluate the humoral immune homeostasis of the body. IgA, IgG, and IgM are the main components of serum antibodies, accounting for about 98% of the total amount of serum antibodies, and the content of antibodies in serum reflects the strength of the immune function of the body (Rea et al., 2018). Further, the effects of saponins on blood IgA, IgM, and IgG were analyzed. The results showed that saponin Rb_1_ and Re administration significantly increased IgA levels in aging models, but had no significant effect on IgM levels. In particular, IgG, Rb_1_, and Re administration dramatically increased its levels. When our body is infected by an organism, our immune system rapidly produces IgA and IgM and acts as the earliest antibodies to defend against or delay the infection. Within a few weeks, our immune system produces IgG, while IgA and IgM gradually break down. The results of this study showed that after 8 weeks of saponin administration, IgG increased significantly, indicating that Rb_1_ and Re can bring long-term infection defense to the body, that is, the immunity has been improved for a long time.

The thymus produces naive CD4^+^ lymphocytes that can differentiate along different pathways, such as Th1 cells that produce interferon INF-γ to fight intracellular infections and tumors (Tittes et al., 2024); Th2 cells can produce interleukin-4 (IL-4 is used to fight worms (Jiang et al., 2021); Th17 cells produce IL-17 to fight off extracellular infections (Mills, 2023). IL-10, the most widely known anti-inflammatory cytokine, is produced by a variety of cell types and by virtually all subpopulations of white blood cells (Kalmarzi et al., 2019). IL-6 is a soluble mediator with pleiotropic effects on inflammation, immune response, and hematopoiesis, it supports the growth of B cells and has antagonistic effects on regulatory T cells (Zhou et al., 2020). IL-2 regulates the activity of white blood cells responsible for immunity. IL-2 mediates its use by binding to the IL-2 receptor expressed by lymphocytes (Ghasemi et al., 2016). The main sources of IL-2 are activated CD4+T cells and activated CD8+T cells. IL-27 is greatly involved in differentiation by inducing or inhibiting each T cell subpopulation, and IL-27 can inhibit Treg cells by regulating the expression of GATA1 and STAT3 (da Silva and Schumacher, 2021). TNF can inhibit the replication of different influenza viruses and has a strong antiviral effect. The main function of TNF is to regulate the function of immune cells. As an endogenous pyrogen, it can promote fever, cause apoptosis, induce sepsis by inducing the production of IL1 and IL6, induce dysplasia, induce inflammation, prevent tumorigenesis and viral replication (Gabuzda et al., 2020). IL-2 is an important immune enhancer, which can enhance the immune function of the body by promoting the proliferation of a specific T cell population (Puissant-Lubrano et al., 2015). IL-2 was significantly reduced in aging mouse models, and the remission effect of Rb_1_ and Re on IL-2 was significant, but the simultaneous administration of Rb_1_ and Re did not show a better effect than that of single administration, indicating that RB_1_ and RE have similar ability to enhance the immunity through IL-2 pathway, and may have a positive effect on T cell population. Alone administration can achieve a strong effect of immune function recovery. Similarly, IL-6 can induce B-cell differentiation to produce autoantibodies, causing the body to produce severe inflammatory responses (Hu et al., 2016). In this study, the IL-6 level of aging model mice was maintained at a high level, which indicated that their immune function had a certain decline. When Rb_1_ and Re were administered, IL-6 levels decreased significantly, suggesting that Rb_1_ or Re had a cumulative effect on the immune system throughout the body. In addition to IL-6, TNF is also an important pro-inflammatory factor. However, the results of this study showed that Rb_1_ and Re had little effect on TNF, and only the simultaneous administration of Rb_1_ and Re had a significant difference. According to the results of this study, we believe that Rb_1_ and Re have positive effects on some pro-inflammatory factors, but not on all pro-inflammatory factors. However, the simultaneous administration of Rb_1_ and Re can produce a significant immune recovery effect. Both Rb_1_ and Re have positive effects on anti-inflammatory factors such as IL-10 and IL-4. In summary, saponins Rb_1_ and Re have positive effects on immunity mainly through two ways, one is to inhibit the level of pro-inflammatory factors, and the other is to increase the level of anti-inflammatory factors. After the administration of Rb_1_ and Re, the pro-inflammatory factors and anti-inflammatory factors achieve a better balance, which maintains a higher immunity of the body.

## 5 Conclusion

Rb_1_ and Re alone can significantly increase the spleen index of aging model, and the apoptotic cells in thymus are slightly reduced, and the proportion of apoptotic cells is decreased. Rb_1_ + Re combined intervention can promote the increase of thymus index and spleen index, which is more advantageous than Rb_1_ or Re alone intervention. Meanwhile, apoptotic cells in thymus are significantly reduced, which indicates that Rb_1_ + Re intervention mode has a alleviating effect on thymus cells in aging state. The combined intervention of saponin Rb_1_ and Re significantly increased IgA and IgG levels in aging mice, but had no significant effect on IgM levels. When Rb_1_ and Re were simultaneously treated, IL-10 levels increased to levels close to Col. In addition, the levels of IL-6, IL-17, and IL-2 were highest in aged mice, but lowest in Col, while their levels decreased significantly after intervention with Rb_1_ or Re. The aged mice had higher levels of interferon, TNF and INF than the control group. After Rb_1_ and Re intervention, INF levels returned to levels comparable to Col, but TNF was not affected. In terms of TNF, simultaneous administration of Rb_1_ and Re can significantly reduce TNF levels in aging model mice. In conclusion, the combined intervention of Rb_1_ and Re can delay the decline of immune system function in aging model, and is significantly better than single intervention.

## Data Availability

The original contributions presented in the study are included in the article/Supplementary material, further inquiries can be directed to the corresponding author.
